# Decision-making without a brain: how an amoeboid organism solves the two-armed bandit

**DOI:** 10.1098/rsif.2016.0030

**Published:** 2016-06

**Authors:** Chris R. Reid, Hannelore MacDonald, Richard P. Mann, James A. R. Marshall, Tanya Latty, Simon Garnier

**Affiliations:** 1 Department of Biological Sciences, New Jersey Institute of Technology, Newark, NJ 07102, USA; 2 School of Mathematics, University of Leeds, Leeds LS2 9JT, UK; 3 Department of Computer Science, University of Sheffield, Sheffield S1 4DP, UK; 4 Department of Animal and Plant Sciences, University of Sheffield, Sheffield S10 2TN, UK; 5 School of Life and Environmental Sciences, University of Sydney, Sydney, New South Wales 2006, Australia

**Keywords:** slime mould, *Physarum polycephalum*, decision-making, exploration–exploitation trade-off, Bayesian model selection, two-armed bandit

## Abstract

Several recent studies hint at shared patterns in decision-making between taxonomically distant organisms, yet few studies demonstrate and dissect mechanisms of decision-making in simpler organisms. We examine decision-making in the unicellular slime mould *Physarum polycephalum* using a classical decision problem adapted from human and animal decision-making studies: the two-armed bandit problem. This problem has previously only been used to study organisms with brains, yet here we demonstrate that a brainless unicellular organism compares the relative qualities of multiple options, integrates over repeated samplings to perform well in random environments, and combines information on reward frequency and magnitude in order to make correct and adaptive decisions. We extend our inquiry by using Bayesian model selection to determine the most likely algorithm used by the cell when making decisions. We deduce that this algorithm centres around a tendency to exploit environments in proportion to their reward experienced through past sampling. The algorithm is intermediate in computational complexity between simple, reactionary heuristics and calculation-intensive optimal performance algorithms, yet it has very good relative performance. Our study provides insight into ancestral mechanisms of decision-making and suggests that fundamental principles of decision-making, information processing and even cognition are shared among diverse biological systems.

## 1. Introduction

While less recognized than their animal counterparts, many non-neuronal organisms, such as plants, bacteria, fungi and protists, also have the ability to make complex decisions in difficult environments (for a full review, see [1]). The most incredible feats of problem-solving among non-neuronal organisms, many previously reported only in the so-called cognitive organisms, have been demonstrated by the unicellular slime mould *Physarum polycephalum*. This unicellular protist lacks a central nervous system and possesses no neurons, yet it has been demonstrated to solve convoluted labyrinth mazes [2], find shortest length networks and solve challenging optimization problems [3], anticipate periodic events [4], use its slime trail as an externalized spatial memory system to avoid revisiting areas it has already explored [5] and even construct transport networks that have similar efficiency to those designed by human engineers [6]. Slime mould cells also display similar decision-making constraints to the cognitive constraints observed in brains. Latty & Beekman [7] provide evidence that *P. polycephalum* is vulnerable to making the same economically irrational decisions that can afflict humans [8], starlings [9], honeybees [10] and grey jays [10]. The same authors also demonstrate that, like humans, slime moulds are subject to speed-accuracy trade-offs when confronted with a difficult choice set [11]. Studies such as these support the growing notion that certain problem-solving processes, as well as their associated trade-offs and paradoxes, are spread wide on the phylogenetic tree [12,13]. To compare the information processing abilities of different organisms, we require a common testing platform based on challenges likely shared by organisms from vastly different taxa.

Many of the decisions faced by humans and most other organisms necessitate exploration of a number of options before a commitment is made to exploit a particular choice. These decisions are often made more complex when their variables are continually changing, resulting in a need for constant re-evaluation of alternatives. The question boils down to a fundamental conundrum in decision-making: to exploit familiar but potentially sub-optimal options, or to risk further exploration for potentially more rewarding ones? This is known as the exploration–exploitation trade-off. Several studies in both humans and other animals have examined the exploration–exploitation trade-off using what has become a classic behavioural experiment in the understanding of decision-making; the multi-armed bandit problem. The multi-armed bandit problem derives its name from casino slot machines—deciding which machine to play to maximize the net payoff proves to be nearly impossible for the average gambler to consistently solve [14]. Only one provably optimal schedule has been derived for the special case of stationary bandit problems where there are no costs for switching between arms—the Gittins index [15]. Empirical studies of bandit problem-solving have thus far only been carried out in organisms with brains (such as humans [16], great tits [17], pigeons [18], sticklebacks [19] and bumblebees [20]). However, the exploration–exploitation trade-off is a problem faced by unicellular organisms as well, which must tackle the problem without the aid of complex nervous systems. Given the sophisticated problem-solving abilities of the slime mould *P. polycephalum*, we chose to examine this protist's decision-making capabilities by challenging slime mould cells with two-armed bandit problems of increasing difficulty. Beginning with a simple, static choice between two arms of different quality, we advanced through levels of difficulty to our most challenging trials in which slime moulds must make decisions in noisy, unpredictable environments. We next uncovered the proximate decision rules used by slime moulds to make ‘good’ decisions. Finally, we used Bayesian model selection to select the most likely behavioural algorithm employed by the slime mould cells, and compared this strategy to the performance expected by the more complex, and in some cases provably optimal algorithms such as the Gittins index.

## 2. Material and methods

### 2.1. Biological material

The vegetative state of *P. polycephalum*, called a plasmodium, is a large, multinucleate cell. The general morphology of a plasmodium includes an extending ‘search front’ at the leading edge of the migrating cell, typically forming a dense fan-shape. This is followed by a system of intersecting tubules towards the trailing edge of the organism. Protoplasm is constantly and rhythmically streamed back and forth through the network of tubules, circulating chemical signals and nutrients throughout the cell (see videos at https://chrisrreid.wordpress.com/labwork/).

We maintained *P. polycephalum* plasmodia on plates of 1% w/v agar with 5% w/v dissolved oat powder (Muscle Feast™ Whole Oat Powder) in the dark at 25°C. We obtained original cultures from Carolina Biological Supply Company^®^, and recultured laboratory stocks on new 5% oat-agar plates weekly.

### 2.2. Shared experimental procedures

**Figure 1.**
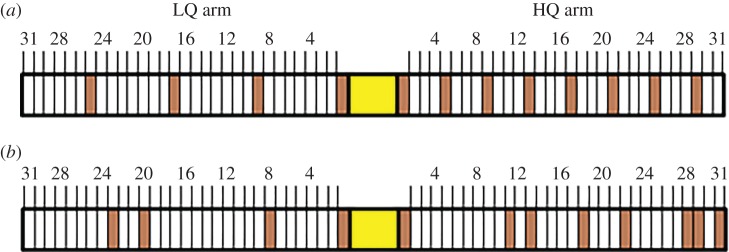
Two-armed bandit experimental set-up for *Physarum polycephalum*. Cell biomass was placed in the centre (yellow box). White boxes indicate blank agar sites (non-rewarding), brown boxes indicate oat-agar food sites (rewarding). Agar sites were 1 mm in diameter. The first site on either arm was always a 5% oat-agar food site, to ensure the cell initialized exploration on both arms. Pictured here are the (*a*) 4e versus 8e treatment, where the LQ arm has evenly distributed reward sites, and the HQ arm has 8 evenly distributed reward sites, and (*b*) 4r versus 8r treatment, where the reward sites were distributed randomly. For graphic representations of other tested distributions, see the electronic supplementary material, figure S1. (Online version in colour.)

To challenge the slime mould with the two-armed bandit problem, we provided *P. polycephalum* plasmodia (the mobile, actively foraging stage of the cell's life cycle) with a choice between two differentially rewarding environments. These two choices constituted ‘arms’ of the two-armed bandit, and differed in their amount and distribution of rewarding food sites (examples provided in figure 1). By expanding pseudopodia equally into both environments, the cell could initially explore both arms. If capable of choosing the better environment, the cell should eventually switch from exploration to exploitation, and continue moving only on the more rewarding arm. We considered the point at which this happens to be where the cell made its decision.

The arms were 31 mm in length, containing varying 1 mm blocks of either 1% w/v blank agar or 1% w/v agar with 5% w/v dissolved oat powder (for all set-ups, see the electronic supplementary material, figure S1). A 6 mm blank agar block was placed between the two arms and acted as the start position for the cells*.* Experiments were set up in lidded Petri dishes. The first block along each arm was always a 5% oat-agar block to ensure exploration of at least one site along each arm. The arm with the greater number of oat-agar blocks was designated the high-quality (HQ) arm, and the other designated the low-quality (LQ) arm. Whether the left arm was the HQ or LQ was randomized between experiments. We placed 0.025 (±0.005) g of plasmodial biomass from our culture on the start block to begin the experiment. This was enough biomass to potentially cover the entire experimental surface area during exploration, ensuring that any differentiation we observed in biomass distribution was due to cell choice, and not constrained by cell size. These cell fragments begin to act as new individual plasmodia within minutes [21]. The time-course of our experiments (up to 48 h) was sufficiently short that any redistribution of biomass was due to active cell movement, not cell growth [22]. The first arm on which the cell reached the last agar block was the arm we considered to be ‘chosen’. The experiment was stopped at this point. Each Petri dish contained two replicates that were isolated from each other by a lack of shared agar substrate.

### 2.3. Data collection and analysis

Each experiment lasted approximately 48 h, with up to 36 replicates at a time. We took photographs every 10 min using a GoPro Hero 3™ camera inside a darkbox. The temperature was maintained at 25°C. An LED panel beneath the Petri dishes provided illumination for photography for a duration of 10 s every 10 min. At all other times, the experiments were kept in darkness. The images were analysed using custom-designed computer vision software (run on Matlab™ version R2014a) that determined the leading edges of the slime mould on each arm. We stopped image analysis after each cell had reached the end of an arm. We excluded any replicates where the cell left the arm and explored the plate before reaching the end of an arm, or invaded or fused with the adjacent replicate on a plate.

For all treatments where one arm was higher in quality than the other, we graphed the proportion of replicates where the cell reached the end of the HQ arm first (‘chose’ that arm). We also produced graphs depicting the dynamics of the decision-making process, by graphing the difference in site discovery between HQ and LQ arms for the first time each site was discovered on either arm.

### 2.4. Choice scenarios

#### 2.4.1. Baseline decision behaviour

As a baseline, we first examined how the cell behaved when given a choice between two environments that were identical in quality. Our treatments thus contained arms that were completely rewarding (31 versus 31), relatively devoid of reward (1 versus 1), or intermittently rewarding in an evenly distributed (8e versus 8e) or randomly distributed (8r versus 8r) pattern (electronic supplementary material figure S1 and see figure S2 for the mean reward site distributions). We compared the cell's behaviour in these treatments to a treatment in which one arm was considerably more rewarding than the other (1 versus 8e).

#### 2.4.2. Consistent reward with dissimilar, regular environments

As a standard bandit scenario, we chose a static, well-structured and predictable exploration environment, where the HQ arm was twice as rewarding as the LQ arm. We therefore set up treatments with an LQ arm of 4 reward sites and a HQ arm of 8 reward sites, distributed evenly along the arms (4e versus 8e, figure 1).

#### 2.4.3. Consistent reward with dissimilar, irregular environments

We next examined how the predictability of the environment affects the decision-making process. Our experimental set-up allowed us to control the pattern of information received by the cell as it explored both environments, simply by controlling the distribution of reward sites along each arm. We were able to ensure that as the cell explored both arms, the information relating to the quality of each alternative could be received in a random manner, representing a more naturalistic and unpredictable environment. We repeated the 4 versus 8 treatment above, but in this case the position of each reward site along the arm was determined randomly. The treatment was thus 4r versus 8r. The mean distributions of reward sites and the randomization method are available in the electronic supplementary material, figure S2.

#### 2.4.4. Sensitivity to reward differences

In many models of decision-making, the level of similarity between options can have a large impact on the decision-making process [23,24]. Our previous treatments used a 1 : 2 ratio of choice quality. Keeping this constant, we first doubled the absolute number of food sites on each arm, in both evenly distributed (8e versus 16e) and randomly distributed (8r versus 16r) scenarios (electronic supplementary material, figure S1). Comparing these treatments with their 4 versus 8 counterparts informs us of the sensitivity of the cell to changes in the absolute quality of the opposing options, while keeping the relative difference in quality constant. We next lowered this relative difference in quality between the arms, such that the LQ arm contained 11 reward sites and the HQ arm contained 16 reward sites (11e versus 16e; electronic supplementary material, figure S1), thereby making the discrimination problem harder. We also repeated the treatment with a random distribution of reward sites (11r versus 16r; electronic supplementary material figure S1. See the electronic supplementary material, figure S2 for distribution of reward sites).

#### 2.4.5. Random, non-binary reward with dissimilar, irregular environments

In the treatments above, all reward sites contained an identical concentration of oats as food (5%). Thus, in evaluating the environments on the LQ and HQ arms, the cell need only compare the number of times a reward has been discovered on each arm. To solve the non-binary two-armed bandit problem, the cell must be able to make the more sophisticated comparison of the magnitude of the rewards returned from each environment sampled. In our next experiments, both arms had an equal number of reward sites (eight) distributed randomly along their lengths. The magnitude of each reward site was chosen randomly between 1% and 8% oat-agar, and the HQ arm contained twice the overall percentage of oat-agar as the LQ arm (reward sites totalling 2.5% oat-agar on the LQ arm and 5% on the HQ arm; electronic supplementary material, figure S1). We refer to this treatment as the ‘non-binary’ bandit. The mean distribution of reward sites is available in the electronic supplementary material, figure S2.

### 2.5. Bayesian model selection

To reveal the specific behavioural algorithm that the cell used in each step of exploring/exploiting the environment, we considered 10 rules of varying complexity which the cell could use to accurately detect the availability of food in the experimental arena and exploit this information to maximize total food intake. We encoded these possible mechanisms as mathematical models for the cell's progression and used Bayesian model selection methods [25,26] to identify which of these models best predicted the observed movements, over all different treatments and the entire duration of the experiments. These models ranged from very simple rules through more complex heuristics, to rules that approximate optimal two-arm bandit algorithms (Thompson Sampling [27]). Our final model was the optimal Gittins process, which provides a benchmark performance level that cannot be exceeded in the bandit problem, assuming a decision-maker with a correct Bayesian prior over alternative environmental states, and extensive computational abilities [15]. The performance of each model was evaluated by comparison to our experimental data (electronic supplementary material, figure S3).

#### 2.5.1. Models

In each case, the model specifies the probability that the cell will move to the right in the next move, *m_t_*, conditioned on its past experiences encoded as six variables; (i) its last previous movement direction, (ii) whether the last movement led to a reward site, (iii) *A*_R_—the number of reward sites it has encountered on the right arm (plus one pseudo-observation); (iv) *A*_L_—the number of reward sites it has encountered on the left arm (plus one pseudo-observation); (v) *B*_R_—the number of non-reward sites on the right arm (plus one pseudo-observation); and (vi) *B*_L_—the number of non-reward sites on the left arm (plus one pseudo-observation). The pseudo-observations account for the effect of a uniform prior distribution on the density of food (between zero and one, see the electronic supplementary material for more information). The following is a summary of the models considered. Where used, *I*(condition) is an indicator variable that takes the value one if the condition is met and zero otherwise. For models 7 and 8, *Q*(*x*, | *A, B*) is a function that represents the rational belief of an agent that food density is *x*, given previous observations *A* and *B* (see the electronic supplementary material for details). For all models, since there are only two arms to sample and cells were observed always to explore, *P*(*m_t_* *=* *L*) = 1 − *P*(*m_t_* *=* *R*).

(1) Autocorrelation: move in the same direction as the previous time step, *P*(*m_t_* *=* *R*) = *I*(*m_t−_*_1_ *=* *R*).
(2) Anti-autocorrelation: move in the opposite direction to the previous time step, *P*(*m_t_* *=* *R*) = *I*(*m_t−_*_1_ *=* *L*).
(3) Most successes: move in the direction where the most reward has been found, *P*(*m_t_* *=* *R*) = *I*(*A*_R_ *>* *A*_L_) + 0.5 *I*(*A*_R_ *=* *A*_L_).
(4) Highest mean: move in the direction with the highest mean number of encountered reward sites, *P*(*m_t_* *=* *R*) = *I*(*A*_R_/(*A*_R_ *+* *B*_R_) *>* *A*_L_/(*A*_L_ *+* *B*_L_)) + 0.5 *I*(*A*_R_/(*A*_R_ *+* *B*_R_) *=* *A*_L_/(*A*_L_ *+* *B*_L_)).
(5) Relative Successes: move with a probability in proportion to the number of reward sites discovered on each arm, *P*(*m_t_* *=* *R*) *=* *A*_R_/(*A*_L_ *+* *A*_R_).
(6) Relative means (Thompson sampling): move with a probability in proportion to the mean number of reward sites encountered on each arm,
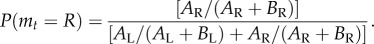
(7) Most likely: move to the arm most likely to have the higher reward density (as estimated from previous reward encounters, see below),
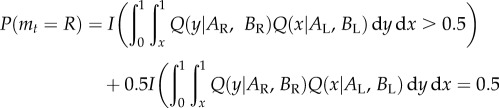
(8) Probability matching: move with a probability that matches the chance of either arm containing the higher reward density,
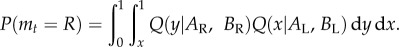
(9) Chemotaxis: our experiments were designed to minimize diffusion of food cues through the agar substrate. Nevertheless, we included a model that accounts for chemotaxis of the cell towards nearby food sites. If *I*_R_ and *I*_L_ are indicator functions for the presence (one) or the absence (zero) of food at the next available position on the right- and left-hand side, respectively, then,
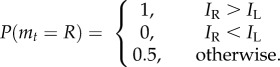
(10) Gittins Index: select the arm with the highest index, which takes account of future expected rewards from both exploration and exploitation of an arm, based on a Beta prior over its expected Bernoulli reward probability, and a discount parameter applied to future rewards. To calculate these we adapted the Matlab™ code from [28], which implements the calibration method for calculating Gittins indices of single-armed bandits with Bernoulli rewards, generalizing this to work for arbitrary hyperparameters of the Beta distribution.

We further incorporated a noise parameter *θ* (detailed in the electronic supplementary material), which represents the proportion of occasions when the cell does not follow the dominant heuristic. We then used the standard procedure of Bayesian performance evaluation via marginal-likelihood, and examined the relative performance of the ‘Relative Successes’ heuristic, both described in further detail in the electronic supplementary material.

## 3. Results

### 3.1. Choice scenarios

#### 3.1.1. Baseline decision behaviour

**Figure 2.**
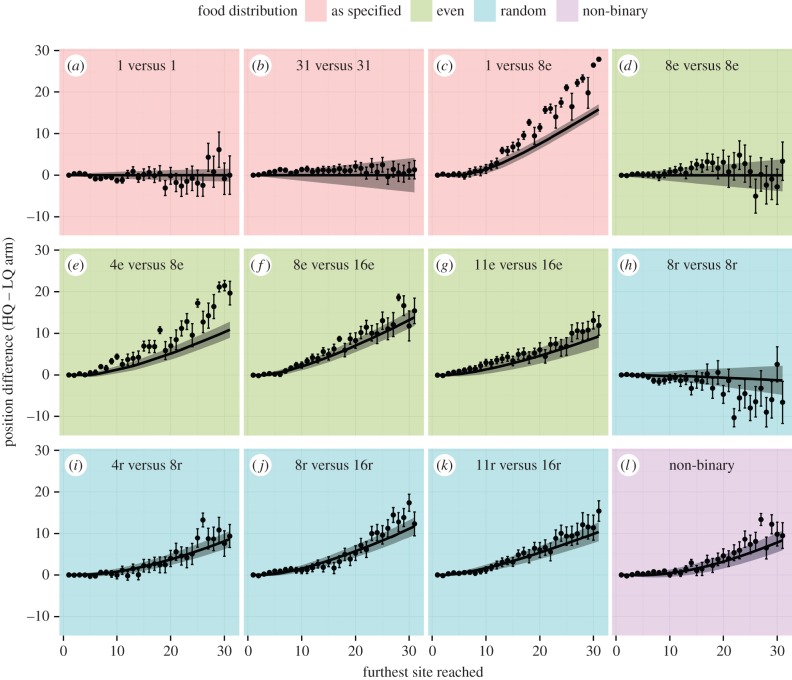
Difference in site discovery between HQ and LQ arms for the first time each site was discovered on either arm. For all treatments, we graphed the difference between the positions of the leading edges of the cell on each arm as each sequential site was first discovered on either arm. That is, whenever the *i*th site was first reached on either arm, the number of sites discovered on the LQ arm was subtracted from the number of sites discovered on the HQ arm. Hence, for treatments where one arm contained a higher reward than the other, positive values indicate more biomass on the HQ arm than the LQ arm, and negative values indicate the opposite. A position difference of zero indicates that both arms were exploited equally. Where both arms were equally rewarding, a positive difference indicates choice of the left arm. Filled circles are experimental data means, error bars are the 95% CIs. Solid lines are the pattern of site discovery predicted by the Relative Successes model, shaded regions are 1.96 s.e. Points where the shaded regions and error bars do not overlap with a position difference of zero indicate a statistically significant preference for the HQ arm (where the difference is positive) or LQ arm (where the difference is negative), at the 95% confidence level. (Online version in colour.)

Regardless of treatment (31 versus 31, 1 versus 1, 8e versus 8e or 8r versus 8r), the cell explored both arms equally, making no decision to exploit one over the other. When one arm was considerably more rewarding (1 versus 8e), the cell chose the more rewarding arm after a short exploration period (figure 2). These results provide the important information that (i) the cell does not make a decision to exploit one environment over another without information suggesting they differ in quality and (ii) the amount of biomass we used per cell was sufficient for the organism to fully exploit both environments simultaneously. Hence, in later experiments when the slime moulds do prefer one environment over the other, they are making a choice to do so. Though the cell is capable of exploring sites on both arms simultaneously, the cell would then ignore the valuable information it has acquired and which should be useful to optimally condition the investment of biomass. Indeed, we only observed simultaneous exploration of sites on both arms in 5% of all timesteps over all of our experiments.

#### 3.1.2. Consistent reward with dissimilar, regular environments

**Figure 3.**
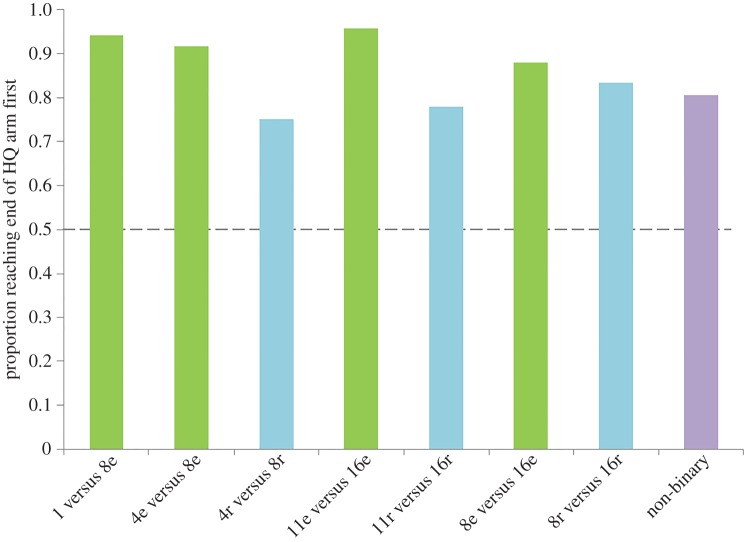
Proportion of replicates in each treatment that reached the end of the HQ arm before they reached the end of the LQ arm. All treatments showed a proportion reaching the end of the HQ arm significantly greater than what would be expected by chance or if the cell could not choose between the two environments (dashed line, 0.5. Binomial test. For *p*-values, see the electronic supplementary material, table S1). Only treatments containing two different quality environments are shown. (Online version in colour.)

As shown in figure 3, the vast majority of replicates in the 4e versus 8e treatment completed exploitation of the HQ environment (reached the end of the arm) first, demonstrating that the cell can choose the better of the two environments. This was the case for all of our subsequent treatments (figure 3), and all relationships were statistically significant (binomial test, electronic supplementary material, table S1). The cells on average displayed a short exploration phase for seven sites on both arms (figure 2), followed by a rapid and exclusive exploitation of the HQ arm. In regular environments, therefore, slime mould appears to undertake a brief period of exploration, followed by exploitation of the most profitable environment discovered.

#### 3.1.3. Consistent reward with dissimilar, irregular environments

When the reward sites were distributed randomly along each arm, the same overall pattern of HQ arm exploitation was observed as for when the reward sites were distributed evenly (figure 2). These results demonstrate that the cell is capable of exploiting the most rewarding arm when information is noisy and obtained randomly. The cell appears to explore for a slightly greater distance (around 11 sites on average) before switching to exploitation than in the above experiments where information was arranged in a regular distribution along the arms (figure 2). Similarly, the difference in exploitation between the arms (figure 2), and the proportion of replicates completing exploitation of the HQ arm first (figure 3), are often slightly higher in the evenly distributed treatments than the randomly distributed treatments. In many randomly distributed treatments, the first few sites of exploration actually presented more reward sites on the LQ arm than the HQ arm. Yet the decision to exploit the HQ arm was made after a similar number of explored sites as when the rewards were distributed evenly above. These results suggest that the cell integrates the quality of each site discovered in the opposing environments over several explored sites, rather than simply responding to the first rewarding site discovered.

#### 3.1.4. Sensitivity to reward differences

The results of the two treatments (4 versus 8 and 8 versus 16) followed identical patterns (figure 2), with a short exploration phase for seven sites on each arm, followed by a rapid and exclusive exploitation of the HQ arm. There was no obvious difference between the evenly and randomly distributed treatments. The choice pattern of the cell indicates that absolute differences between two options are not as important as their relative difference in quality—the cell takes into account the unique qualities of the alternatives available to it, and chooses the better of the two. Previous choice experiments in *P. polycephalum* decision-making have shown that the cell is capable of making a relative comparison of the quality of two or three food sources provided simultaneously [7,11,29]. However in our experiments, the cell was required to integrate the amount of food present over multiple distant sites and discovered at different times in the exploration process, in order to determine the better of two foraging environments. The reduction in relative difference in quality (11 versus 16) did not result in an extended exploration period; however, the overall difference in exploitation was slightly lower than in the other treatments (figure 2).

#### 3.1.5. Random, non-binary reward with dissimilar, irregular environments

Even for our most complicated choice scenario, the pattern of exploration/exploitation was similar to those reported above; a period of exploration of both arms extending to around 12 sites, followed by rapid exploitation of the HQ arm (figure 2). This simple pattern belies a sophisticated and complex problem-solving capability for this protist. Taken together, our results demonstrate that *P. polycephalum* is able to integrate the total food quantity and quality in two randomly provisioned environments, in order to swiftly and accurately predict which environment will provide the most resources for future growth.

### 3.2. Bayesian model selection

The model selected with the highest marginal-likelihood was ‘Relative Successes’; *P*(*m_t_* = *R*) = *A*_R_/(*A*_L_ + *A*_R_), in which the probability of exploring each arm (e.g. *P*(*m_t_* = *R*) for the right arm) is proportional to the number of successes (rewards) previously encountered on that arm (e.g. *A*_R_ for the right arm; electronic supplementary material, figure S3). Figure 2 compares the performance of Relative Successes to the performance of the slime mould for each choice scenario (the relative performance of the other models is provided in the electronic supplementary material, figures S5–S13). Importantly, the decision-making heuristic ‘Chemotaxis’ performed quite poorly in comparison, providing strong evidence that the recent experience of the cell is the information driving decision-making, and not solely chemotaxis towards the arm with the highest reward.

The Relative Successes strategy invokes a level of sophistication far greater than many of our proposed strategies, yet is computationally simpler than the ‘optimal’ strategies such as Thompson Sampling and the Gittins Index. As shown in the electronic supplementary material, figure S4, this strategy still performs well relative to the best achievable performance. Furthermore, the Relative Successes heuristic can be employed in a fully decentralized manner at the local level in the cell by reinforcing exploitation in HQ areas (as in [6]), so it does not require complex global processing based on calculations of either arm being the best. Nonetheless, this strategy performs well in identifying and exploiting the arm with the highest reward, as shown in our experiments and simulations.

## 4. Discussion

The capacity to solve the two-armed bandit problem has previously only been demonstrated in animals with brains. Human subjects have repeatedly been tested with the multi-armed bandit problem and are usually deemed to operate sub-optimally. It is commonly thought that human subjects tend to naturally allow for the possibility that reward rates on the different arms change over time (termed a ‘restless bandit’). Hence, humans tend to switch between exploration and exploitation, and rarely maximize their reward by exclusively exploiting the HQ option [30]. Pigeons [31], great tits [17] and stickleback fish [19] have been shown to learn to exclusively exploit the HQ option in a two-armed bandit scenario. Besides using biological subjects with sophisticated nervous systems, these previous studies all recorded an increase in efficiency gained through repeated testing, and hence learning on behalf of the subject. Our experiments were not specifically designed to test for the effects of learning, in contrast to the previous animal studies; slime mould cells in our experiment were each tested a single time, and so could not learn from past testing. Therefore, the efficiency of the slime mould's strategy described in our results is the result of evolution, rather than individual experience. In the future, it could be interesting to investigate whether repeated testing with *P. polycephalum* leads to an increase in efficiency through learning, given their documented abilities to predict occurrences of events [4].

The non-human animals tested with the multi-armed bandit problem in previous studies [17,19,31] performed close to the optimal rate predicted by the models proposed by the authors. These previous studies only compared their empirical results to models based on economic optimality, whereas in our study we also chose models that tempered pure optimality measures with reasonable biological constraints within which the tested system should operate. The ultimate reason why such a problem-solving capacity might be necessary for a unicellular organism seems clear. The natural foraging environment of *P. polycephalum* is the forest floor, where its prey resources of fungi, bacteria and decaying vegetable matter are distributed patchily [32]. The amoeboid form and large size (potentially exceeding 930 cm^2^ [33]) of the slime mould results in a large area of the environment which can be sensed and explored simultaneously. The ability to quickly compute which areas of the foraging environment will lead to the highest nutritional payoff, and to abandon all areas less profitable, should result in increased fitness and hence be favoured by natural selection.

Without a brain or even neurons, what physical or biochemical mechanisms could be responsible for slime mould decision-making? The slime mould possesses a unique, coupled-oscillator based sensorimotor system that may be the key to its highly developed problem-solving abilities. The cell is composed of many small units, each oscillating at a frequency dependent upon both the local environment and interactions with neighbouring oscillators [34]. When one of these units senses attractants such as food, it oscillates faster, stimulating neighbouring units to do the same, and causing cytoplasm to flow towards the attractant [7]. The reverse process is initiated when repellents such as light are perceived. The collective behaviour of these coupled oscillators, each passing on information to entrain its neighbours, is the most likely platform of decision-making.

The majority of models of decision-making have focused on how neurons in the vertebrate brain interact to reach a decision [35–37]. The central mechanism behind most of these models is the notion that ‘evidence’ in favour of each alternative, in the form of firing rate, builds in competing neurons until a decision threshold is reached [35]. The interaction of competing oscillators in distant regions of the cell may form an analogous function in the slime mould. Evidence in favour of each environment is sensed through the cell membrane and influences the local oscillation pattern. The local oscillation pattern influences the width of transport tubules, and hence controls the flow of protoplasm [34]. Distant oscillators entrain to each other's frequencies, leading to interactions that may influence the final decision and the rate at which it is reached, providing a potential analogy to models of human brains [11]. Similarities in the fundamental principles of such vastly different decision-making systems as human brains, slime mould, and social insect colonies have recently come to the attention of researchers [1,7,38–40]. These similarities raise the compelling notion that deep principles of decision-making, problem-solving and information processing are shared by most, if not all, biological systems. Our framework is a tool for the comparative study of information processing between species and indeed across nearly all taxa.

The advanced problem-solving capacity of the slime mould, at a level previously demonstrated only in brained organisms, provides support for the view that many ‘lower’ organisms can perform cognition-like feats in the absence of a nervous system (often termed ‘minimal cognition’ [41–44]). Intelligence, perception and traditionally higher order cognitive processes are understood to be derived from sensory-motor coupling [41,45]. Classic models separate the ‘lower’ and ‘higher’ organisms by the flow of sensorimotor information processing between the organism and its environment [46,47]; non-cognitive organisms are defined by their reaction to external stimuli without internal feedback between the stimulus receptor and the site of action. By contrast, cognitive organisms modulate the receptor by internal neural feedback from the site of action [46,47]. More recent models of cognition argue that there are many alternative sensorimotor systems that may replace the function of the nervous system in cognition. For instance, van Duijn *et al*. [41] argue that the two-component signal transduction system of the bacterium *Escherichia coli* is a functional sensorimotor equivalent of a nervous system. According to classic cognition models, the oscillation system of *P. polycephalum* may also be a sensorimotor analogue of a nervous system; as information is transferred throughout the cell along the oscillating membrane, oscillators provide internal feedback to each other, and modulate each other's actions. Our results show that taking a wider, more inclusive view of cognition allows a greater appreciation for the broad diversity of information processing, problem-solving and decision-making strategies spread across all taxa.

## Supplementary Material

Revised Supp Mat (third submission).pdf

## Data accessibility

The raw data are deposited in an Open Science Framework online repository, which can be downloaded via this link; https://osf.io/c4mbk/?view_only=36ef8f82e0104a83be1625e9762fa4d5.
