# Supplementary material for: Decision-making without a brain: how an amoeboid organism solves the two-armed bandit
Source: J R Soc Interface. 2016 Jun;13(119):20160030. doi: 10.1098/rsif.2016.0030 (PMC4938078; doi:10.1098/rsif.2016.0030)
Supplement: Revised Supp Mat (third submission).pdf [file rsif20160030supp1.pdf]

Supplementary Material

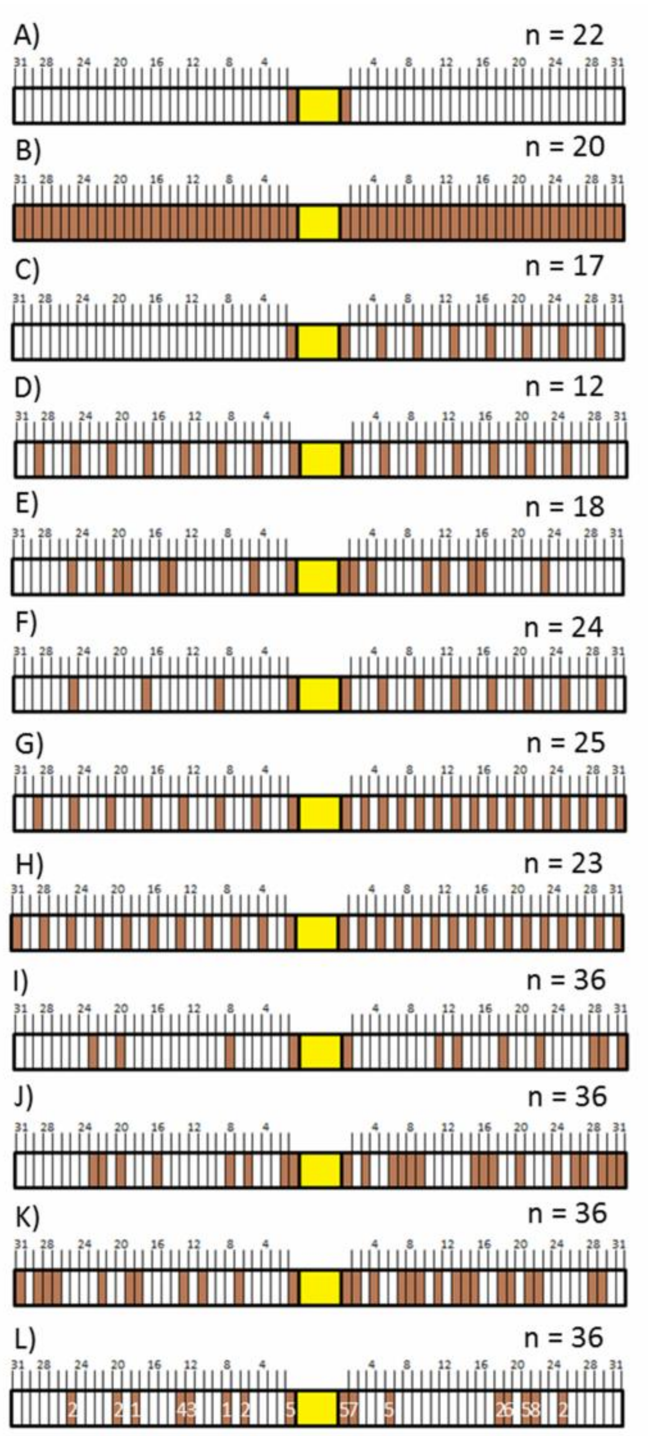

Fig. S1: Experimental setups for the different treatments. *Physarum polycephalum* biomass was placed in the center (yellow box). White boxes indicate blank agar sites

(non-rewarding), brown boxes indicate oat-agar food sites (rewarding). The first site on either arm was always a 5% oat-agar food site, to ensure the cell initialized exploration on both arms. A) 1 vs 1, B) 31 vs 31, C) 1 vs 8e, D) 8e vs 8e, E) 8r vs 8r (single example), F) 4e vs 8e, G) 8e vs 16e, H) 11e vs 16e, I) 4r vs 8r (single example), J) 8r vs 16r (single example), K) 11r vs 16r (single example), L) 'non-binary' bandit (single example). Food sites were 5% oat-agar except in L) (see text), where the numbers give the percentage oat-agar in each reward site for a single example. See Fig. S2 for full details of the random distributions used in the experiments.

Random distributions

The locations of reward sites were selected by random sampling without replacement (using the sample() function in R 3.2.0) from a list of all available sites. We repeated this procedure independently for each arm.

When the magnitude of the reward sites was randomized as well, we followed the following procedure (independently for each arm) to determine the value of each reward site:

1. As for randomly-distributed, equally-rewarding sites, the locations of randomly-distributed, unequally-rewarding sites were selected by random sampling without replacement.
2. All reward sites were allocated a minimum reward magnitude (1% oat-agar). All non-reward sites were kept empty (blank agar).
3. We selected one of the reward sites by random sampling with replacement (using the sample() function in R 3.2.0). This site was given an extra 1% oat-agar, unless

it had already reached the maximum reward magnitude allowed for a single food site (8% oat-agar).

4. We repeated step 2 until we reached the total amount of food reward allocated to the arm.

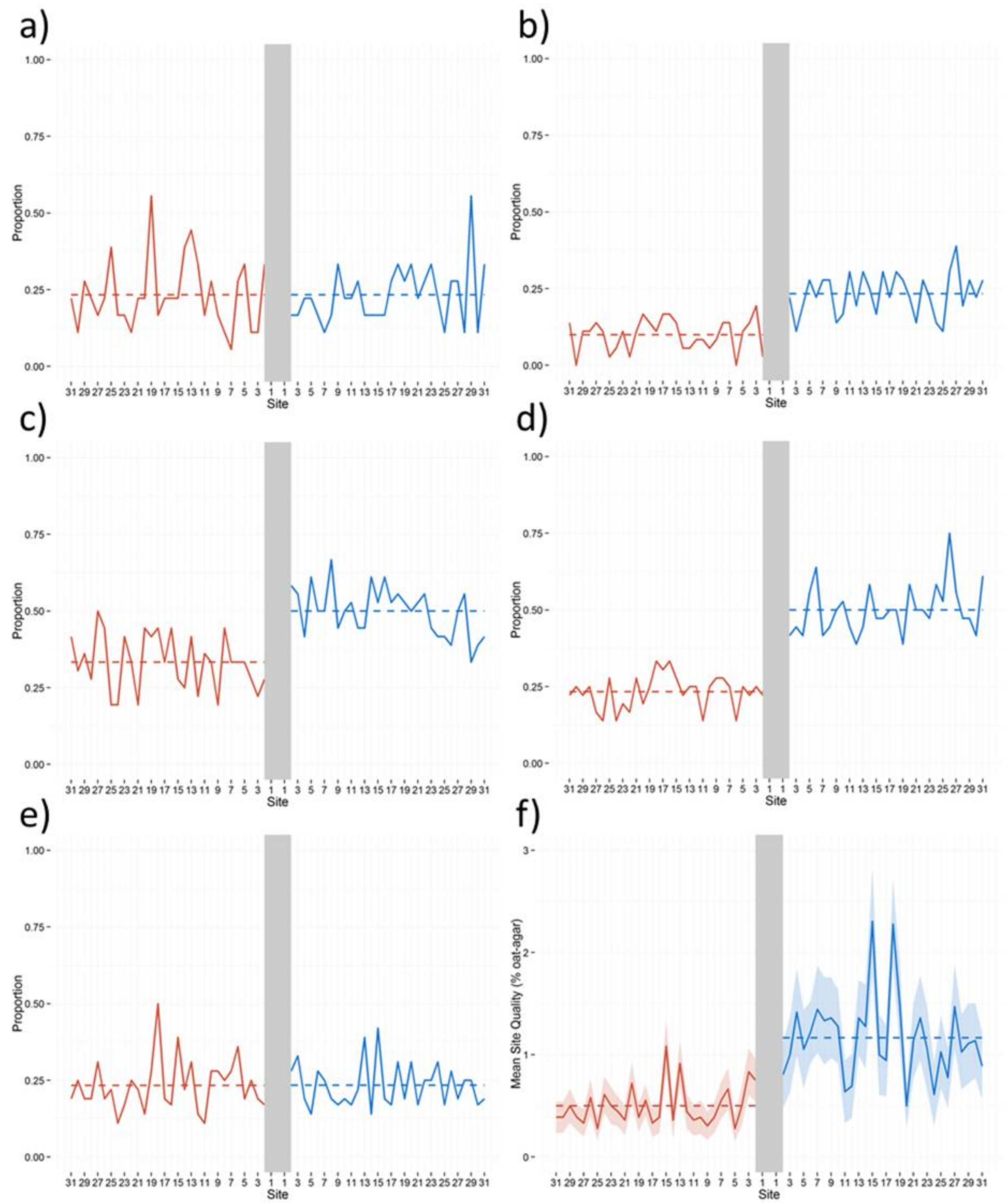

Fig. S2: Proportion of times each site contained reward for treatments; a) 8r vs 8r; b) 4r (red) vs 8r (blue); c) 11r (red) vs 16r (blue); d) 8r (red) vs 16r (blue); e) non-binary bandit, 2.5% arm (red) vs 5% arm (blue). The x axes of the graphs are laid out as if the

reader were looking at a bandit replicate, with the start block in the center (grey bar) and 2 opposing arms of 31 agar blocks, each beginning at site 1 and expanding out to site 31. The start block at site 0 and the first sites on either arm (which were always 5% oat-agar) were excluded from the graphs (greyed bar). The dashed lines show the mean proportion over the entire arm. Mean site quality over all replicates for the non-binary bandit, 2.5% arm (red) vs 5% arm (blue), is shown in f). The solid line shows the mean oat-agar percentage for each site over all replicates. The shaded regions indicate standard error about the mean. The overall mean site quality for each arm is less than 2.5% and 5% because this calculation of the mean includes the blank agar sites on each arm.

Table S1: Binomial test for proportion of replicates reaching the end of the HQ arm first, with the alternative hypothesis that the proportion will be due to random chance (0.5).

Treatment	Proportion reaching end of HQ arm first	p-value
1 vs 8e	0.94	**2.75 x10^{-4}**
4e vs 8e	0.92	**3.59x10^{-5}**
4r vs 8r	0.75	**3.93x10^{-3}**
11e vs 16e	0.96	**5.72x10^{-6}**
11r vs 16r	0.78	**1.19 x10^{-3}**
8e vs 16e	0.88	**1.57 x10^{-4}**
8r vs 16r	0.83	**6.96x10^{-5}**
Non-binary	0.81	**3.13 x10^{-4}**

Rational beliefs about food density

Models 7 and 8 (see main text) depend on the cell's current rational 'belief' about which arm contains the highest density of food, based on the observed successes and failures, along with the prior beliefs. Focusing on a single arm of the experiment, we specify as Q(x | A, B) the probability (from the perspective of the cell) that the food density on that arm is x, conditioned on the cell's number of previous encounters with food-filled or empty blocks on that arm. We condition on two parameters, A and B, to denote how the cell 'learns' from experience. To determine the rational belief Q(x | A, B) we used a simple Bayesian update rule, based on a binomial likelihood function for receiving reward and a uniform flat prior on reward densities. We assign a uniform prior probability over all possible food densities before the cell has any experimental experience.

$$P(\text{Food density} = \text{x}) \equiv \text{Q}(\text{x} \mid \text{A}{=}1, \text{B}{=}1) = 1 \ \forall\ 0 \leq \text{x} \leq 1$$

This flat prior can be expressed as a Beta distribution with parameters A = 1, B=1 (hence our use of these parameters in Q). The Beta distribution is a conjugate prior to the binomial likelihood, which specifies the likelihood (again from the cells perspective) of each new encounter with either food blocks or empty blocks. By the rules of conjugate updating, via Bayes rule, when the cell encounters blocks with or without food it will update its probability distribution over x to a Beta distribution with different parameters, adding one to A for each 'success' (encountering food) and adding one to B for each 'failure' (encountering an empty block). Using this prior distribution therefore means

that once the cell has experienced (A-1) food blocks and (B-1) empty blocks on the focal arm, its rational belief about the food density will be:

$$Q(x\,|A,B) = \beta(x|A,B) = \frac{x^{A-1}(1-x)^{B-1}}{\int_0^1 x^{A-1}(1-x)^{B-1}\,dx}$$

To determine, for instance, the rational belief that the food density, *y*, on the right arm is greater than the food density, *x*, on the left, we simply calculate all the ways this could be true based on the current Q's for each arm.

$$P(y > x) = \int_0^1 \int_x^1 Q(y|A_R, B_R)\, Q(x|A_L, B_L) dydx$$

Noise

We did not expect that the cell would follow any of these rules absolutely faithfully at all times. To account for this variability we introduced a 'noise' parameter, θ, such that the rule is followed with probability (1-θ), otherwise with probability θ the cell chooses a direction at random;

P(m_t = R | θ) = (1-θ) P(m_t = R) + θ/2

Bayesian performance evaluation via marginal-likelihood

The performance of each model was evaluated by calculating the marginal likelihood of all observed moves, across all experiments and all treatments, accounting

for the ‘noise’, θ - the proportion of times that the cell makes a random choice rather than following the rule. Summing over possible values of θ, the marginal likelihood of a model M is thus given as;

$$P(D|M) = \int_0^1 \prod_{Experiments} \left[\prod_{moves\ left} \big((1-\theta)P(L|M,X) + 0.5\theta\big) \prod_{moves\ right} \big((1-\theta)P(R|M,X) + 0.5\theta\big) \right] d\theta$$

where X indicates all the information the cell has from its previous movements, and D represents the data of all observed movements.

Fig. S3 shows the marginal likelihoods of each model both on the full data set of all treatments, and for each treatment individually, normalized by the number of observed movements to indicate the geometric mean probability assigned to each movement. Values greater than 0.5 indicate that the model is predicting better than a null model where each movement is left or right with equal probability. The ratio of the marginal likelihoods of two models is termed the Bayes factor, Jeffreys (1) provides a scale for qualitative interpretation of Bayes factors, for example considering a Bayes factor of 3 to indicate ‘substantial’ evidence for model i over model j, and a Bayes factor of over 100 to be ‘decisive’. Quantitatively, the Bayes factor requires no reinterpretation and simply represents the relative probability that each model is correct. The Bayes factor between our best performing model (‘Relative Successes’) and its nearest competitor exceeds 10^{13},

and therefore the evidence is clearly decisively in favor of this model from among those we have tested.

The Gittins algorithm contains a parameter, gamma, which controls the discount on possible future rewards. This is unique to the Gittins model and is not included in any of our heuristics. We wanted to be certain that the observed decisions of the cells could not be the result of applying the optimal Gittins algorithm, rather than one of our heuristic models. As such we gave the Gittins model (model 9) the freedom to select a discount parameter that best-fitted the observed decisions, rather than performing a Bayesian marginalization. Thus we were able to show that no set of parameters for the Gittins algorithm were able to predict the observed decisions better than our best-performing heuristic, 'Relative Successes'.

The results in Fig. S3 represent marginal likelihood evaluated on the basis of a uniform prior belief attributed to the cell; that is we assumed that before it observed any information about the food distribution the cell 'believes' that the food density is equally likely to be anywhere between 0 and 1. We relaxed this assumption by testing a wide range of other initial beliefs and found in all cases that the ordering of the models was unaffected. In particular 'Relative Successes' continued to outperform all other models by a large margin.

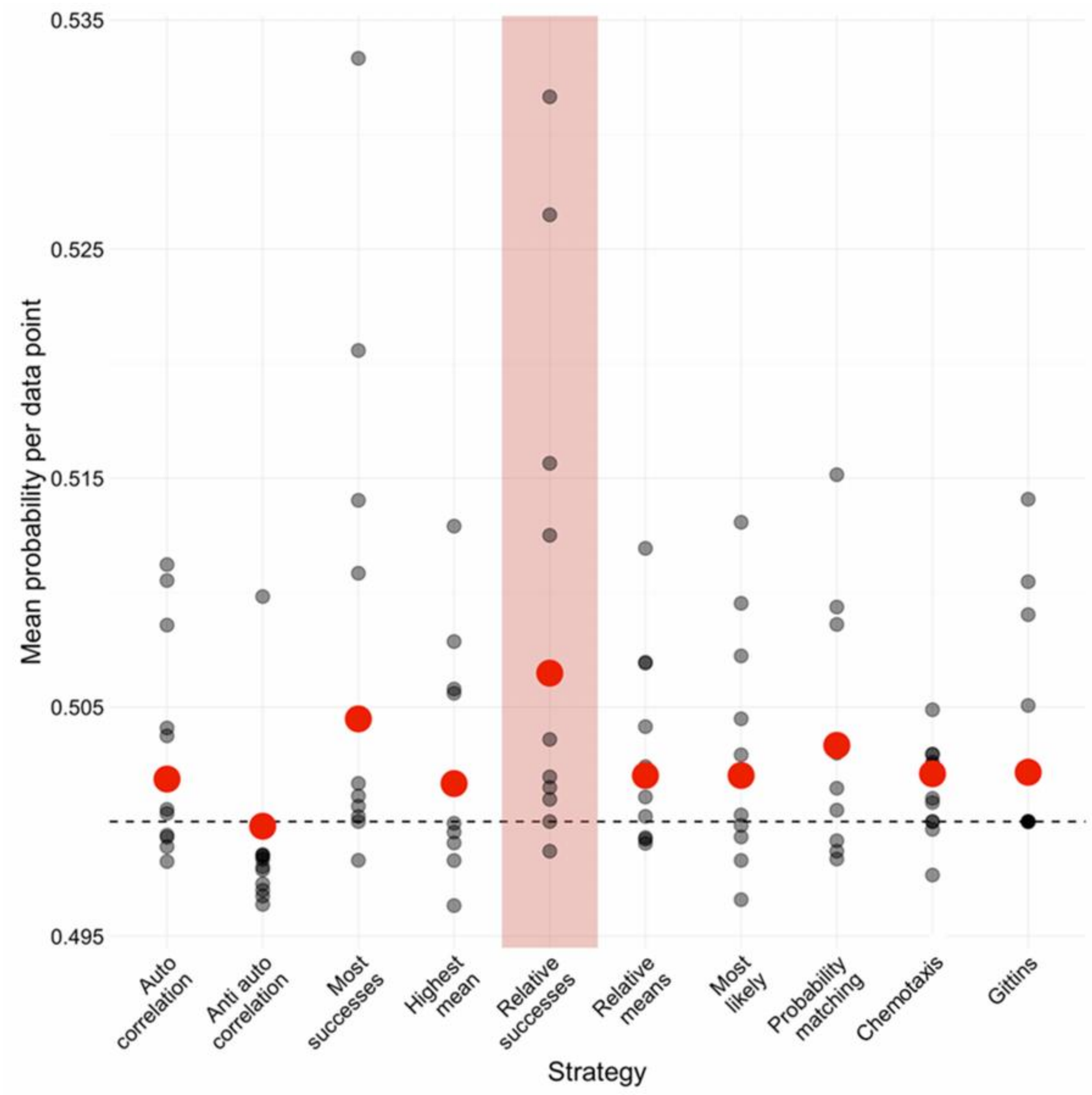

Fig. S3: Bayesian comparison between the proposed models. After calculating the marginal-likelihood of the observed decisions for each model, we normalized by the number of decisions to obtain the probability-per-decision. A value of 0.5 indicates the equivalent of a random prediction, thus values above 0.5 perform better than the 'Random' model. Grey points indicate the results from the eleven different treatments for each model, while the larger red points indicate the values from all of the treatments combined. Overall the highlighted 'Relative Successes' model is strongly favored.

Relative Performance of Relative Successes Heuristic

To benchmark the performance of the relative successes heuristic we compared its expected performance against the expected performance of random choice, and of the optimal Gittins index policy, for a variety of hyperparameters of the beta-distributed Bayesian prior (α and β), and discount rate over future rewards ($1-\gamma$), according to the equation;

relative performance = (heuristic performance - random performance) / (Gittins performance - random performance),

where we assume that the decision maker is equipped with the correct prior for the environment it is situated in. Sample relative performances are plotted in Fig. S4, which shows that as discount rate decreases (γ increases and future rewards become more important) the relative successes heuristic becomes closer to optimal for a larger range of possible priors. At $\gamma = 0.9$ the relative successes heuristic's performance is half way between random performance and the maximum possible performance as realized by the Gittins index strategy for almost all priors studied.

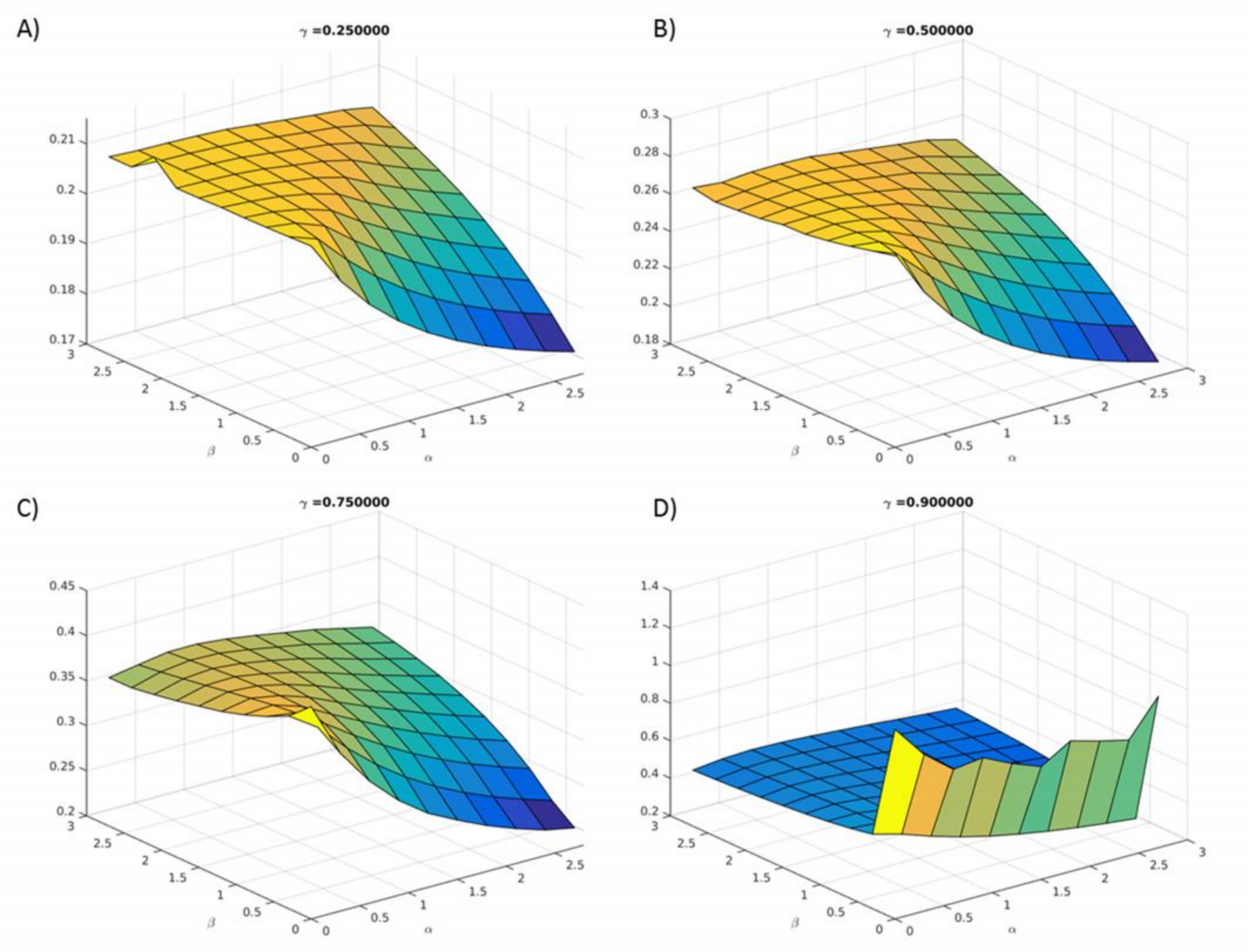

Fig. S4: Sensitivity of best-fitting heuristic's relative performance (0 = random performance, 1 = optimal performance calculated according to Gittins index policy) to variation in hyperparameters of the beta-distribution describing food distribution (α and β) and discount rate applied to future rewards (γ). As the future becomes more important (γ increases) performance of the best-fitting heuristic relative to the theoretical optimum increases. With the exception of γ=0.9 highest relative performance is achieved when β>α, which corresponds to food distributions in which each foraging step is more likely to result in no food than in the discovery of food, suggesting the ancestral environments in which the heuristic evolved may have had this feature.

Relative Performance of Other Heuristics

The following figures show difference in site discovery between HQ and LQ arms for the first time each site was discovered on either arm in each choice scenario (as in Fig. 2). Filled circles are experimental data means, error bars are the 95% confidence intervals. Solid lines are the pattern of site discovery predicted by particular heuristic, shaded regions are 1.96 standard errors.

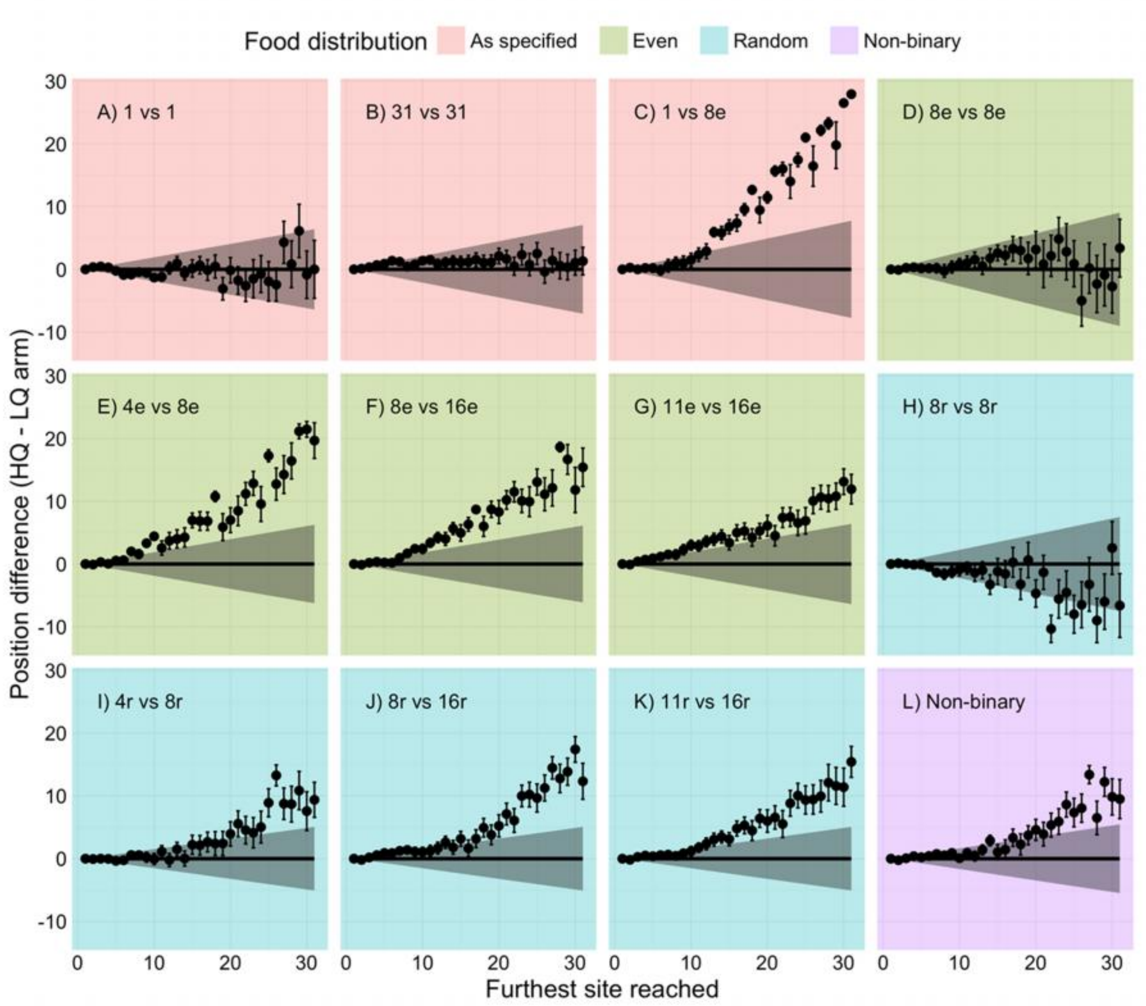

Fig. S5: Relative performance of ‘Autocorrelation’ heuristic.

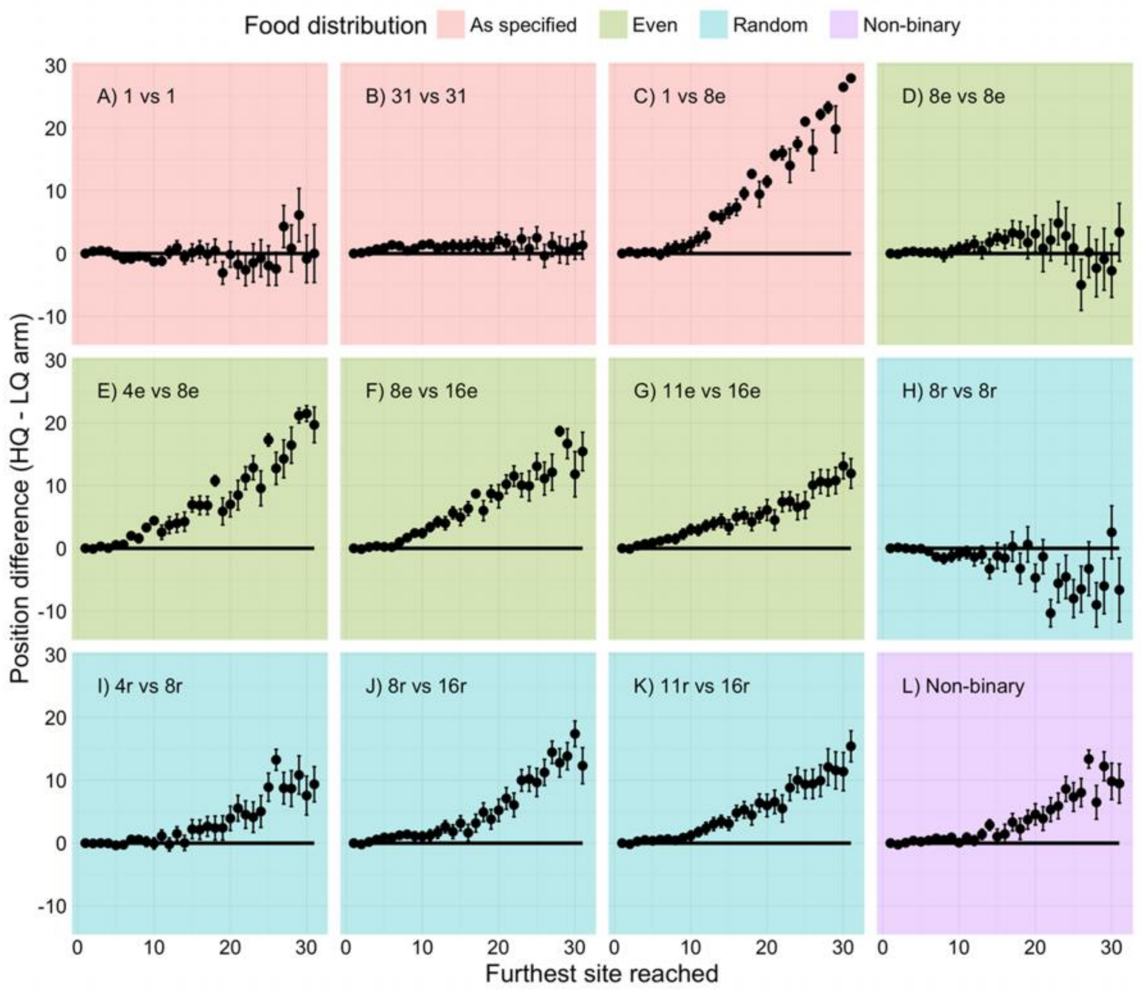

175

176 Fig. S6: Relative performance of ‘Anti-autocorrelation’ heuristic.

177

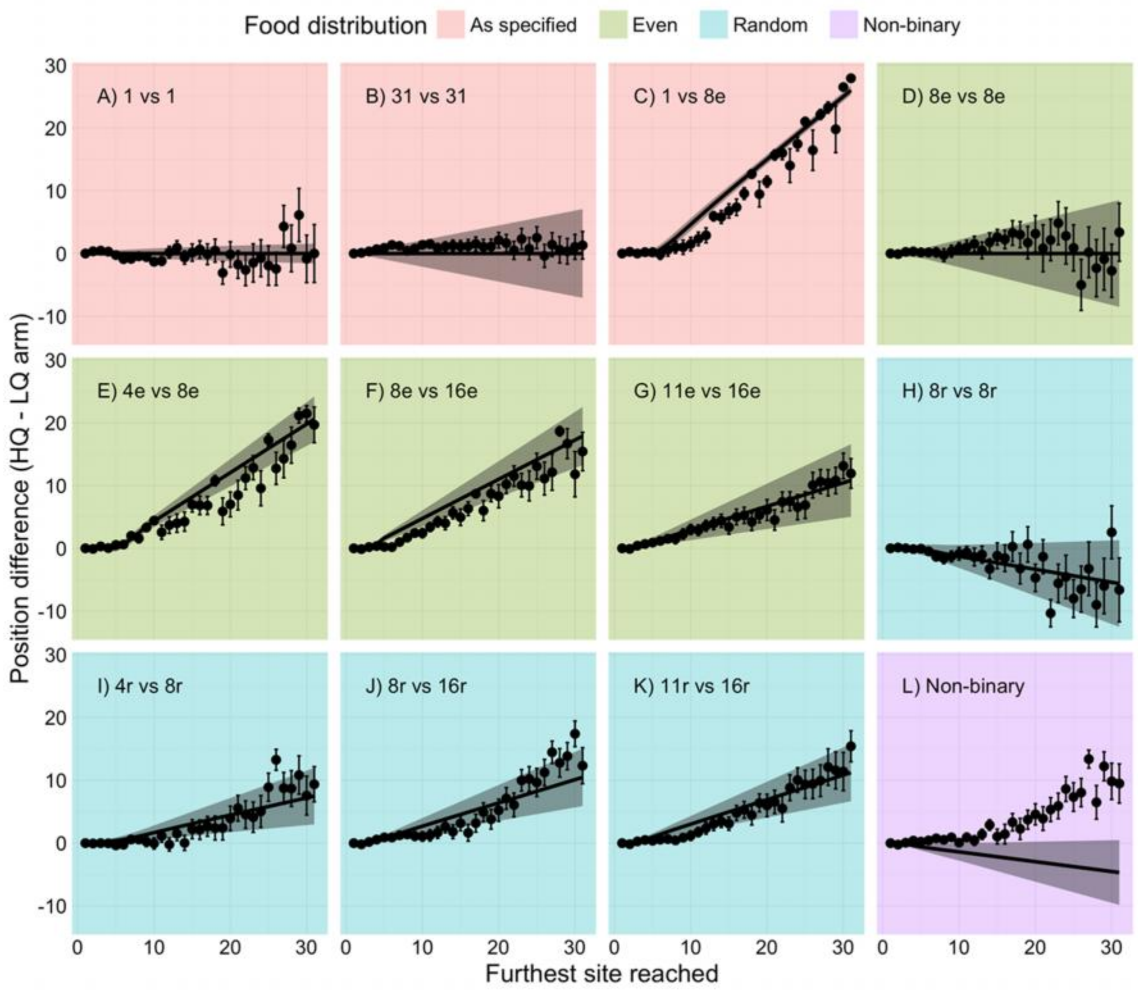

Fig. S7: Relative performance of ‘Most successes’ heuristic.

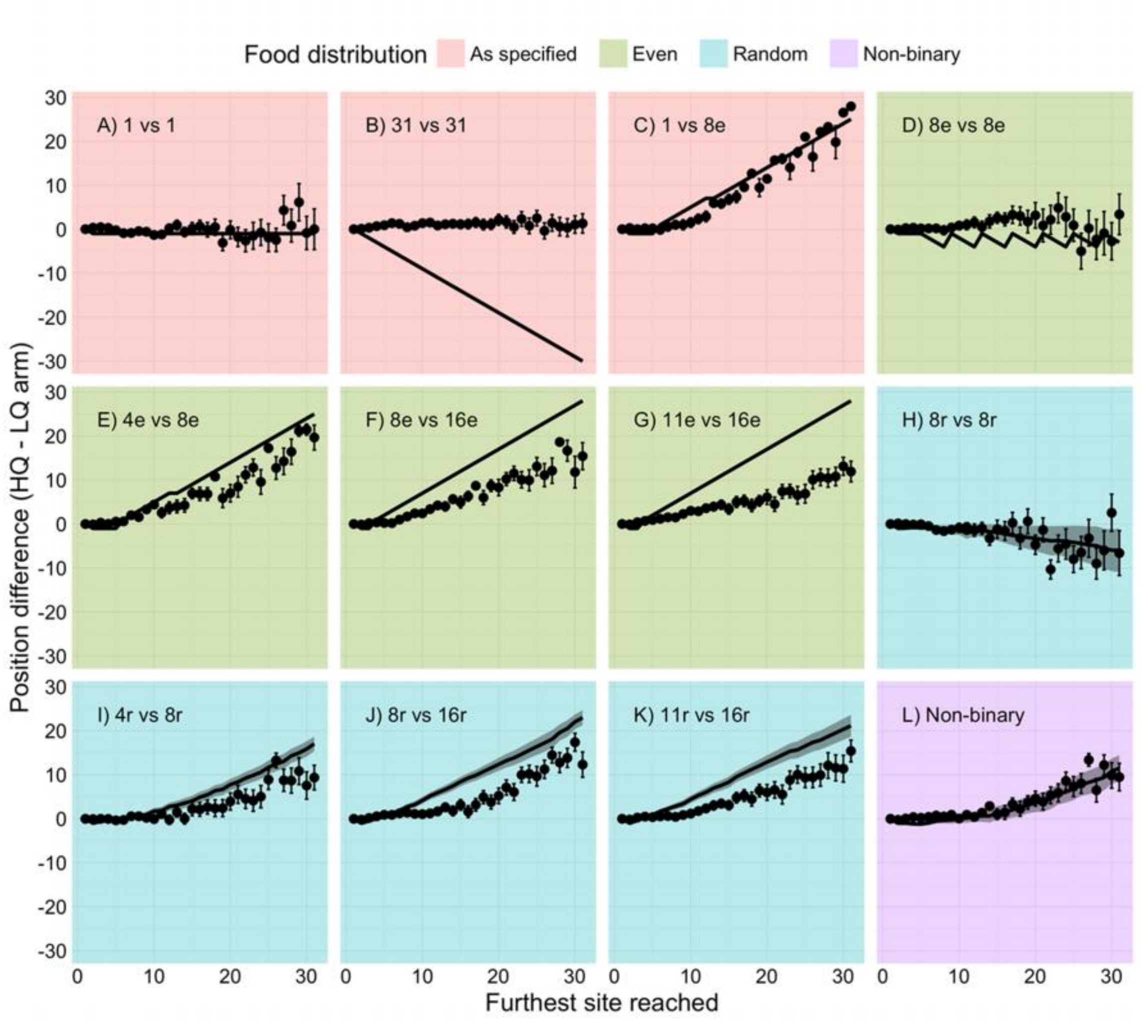

181

182 Fig. S8: Relative performance of 'Highest mean' heuristic.

183

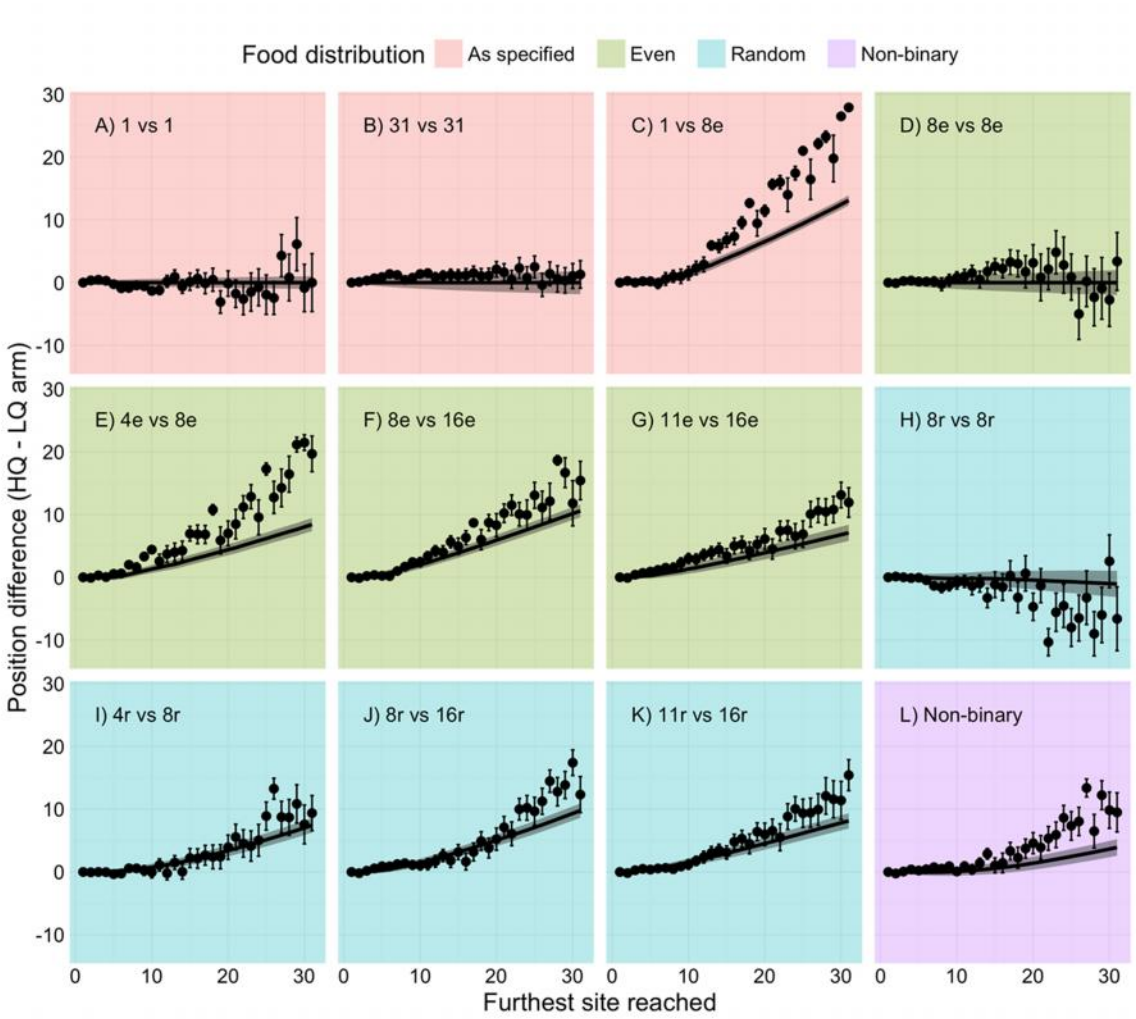

Fig. S9: Relative performance of ‘Relative means’ heuristic.

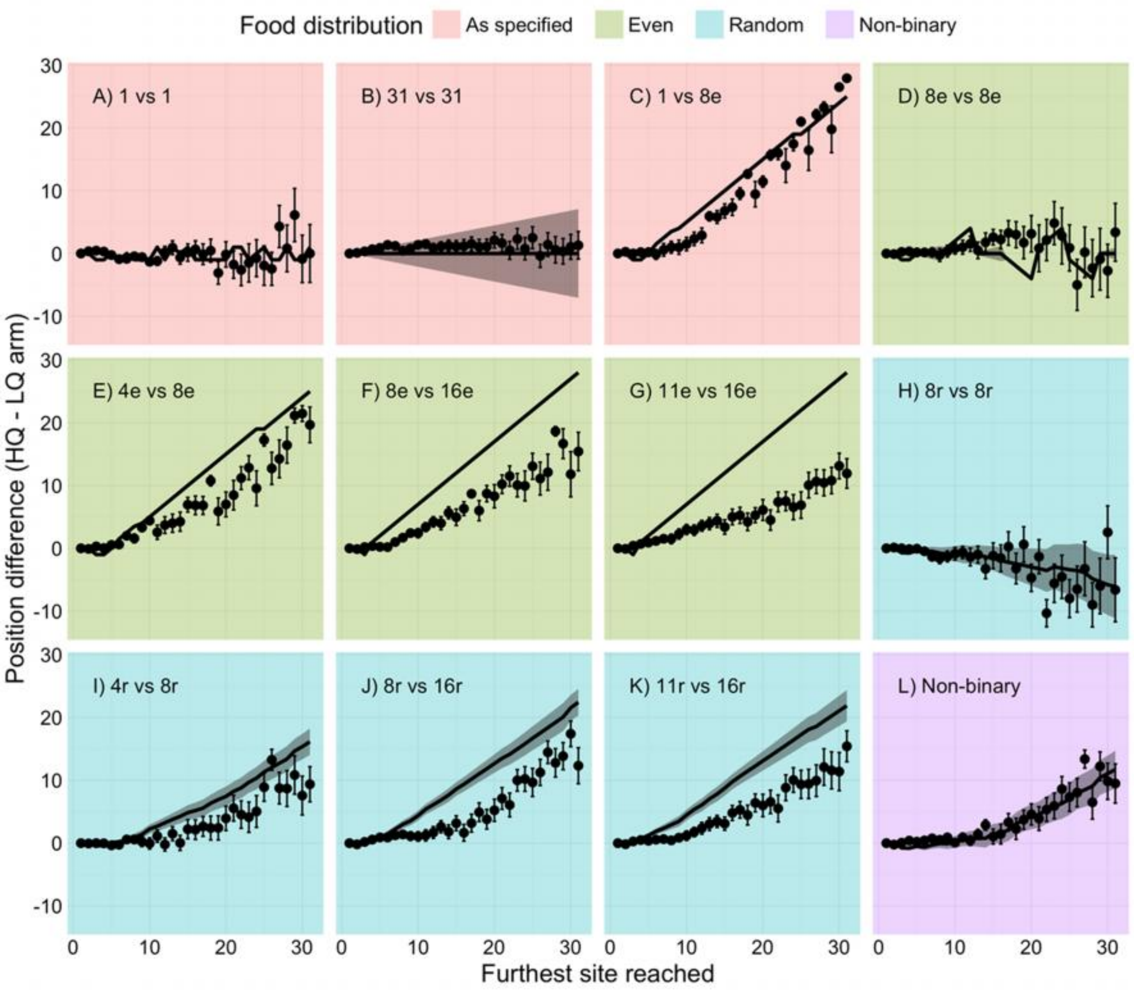

Fig. S10: Relative performance of ‘Most likely’ heuristic.

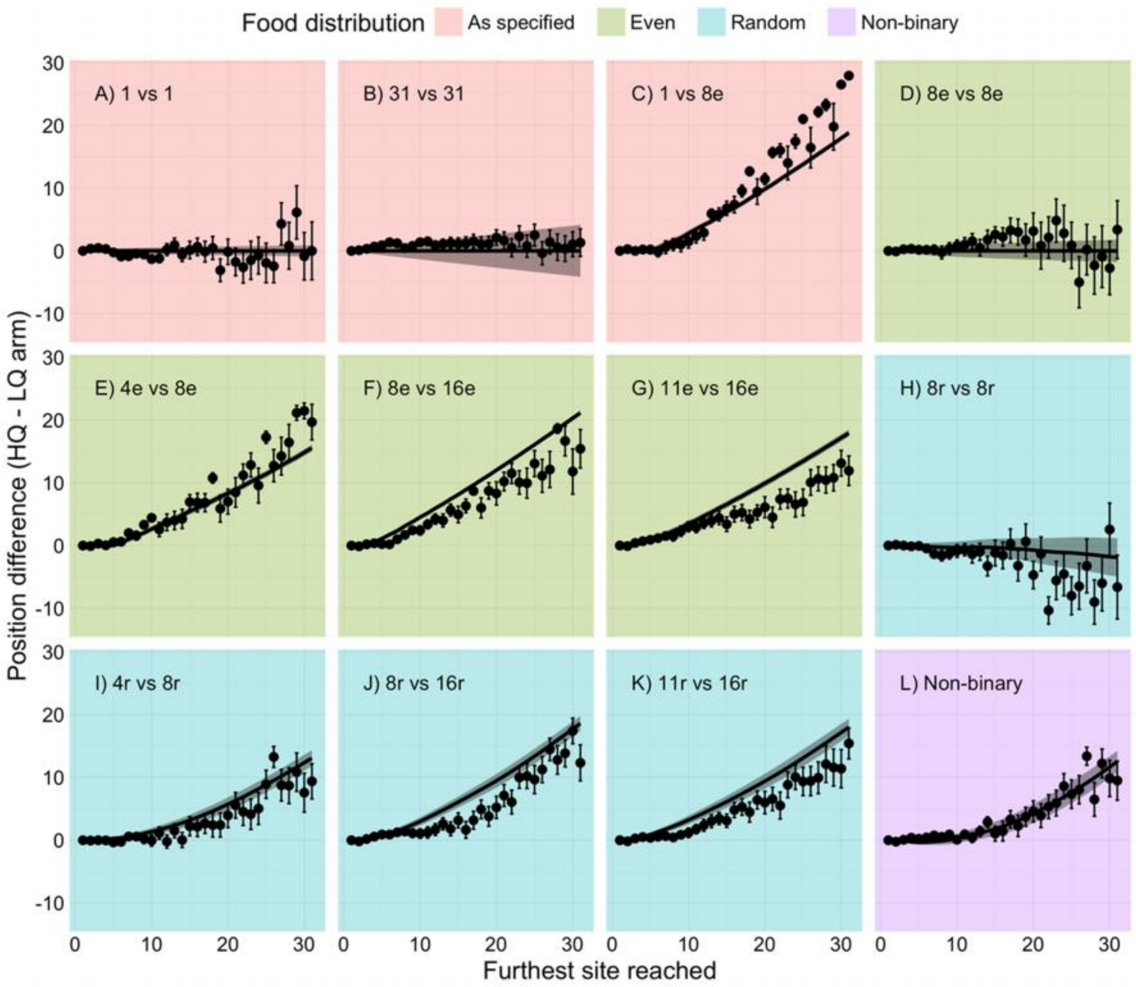

Fig. S11: Relative performance of ‘Probability matching’ heuristic.

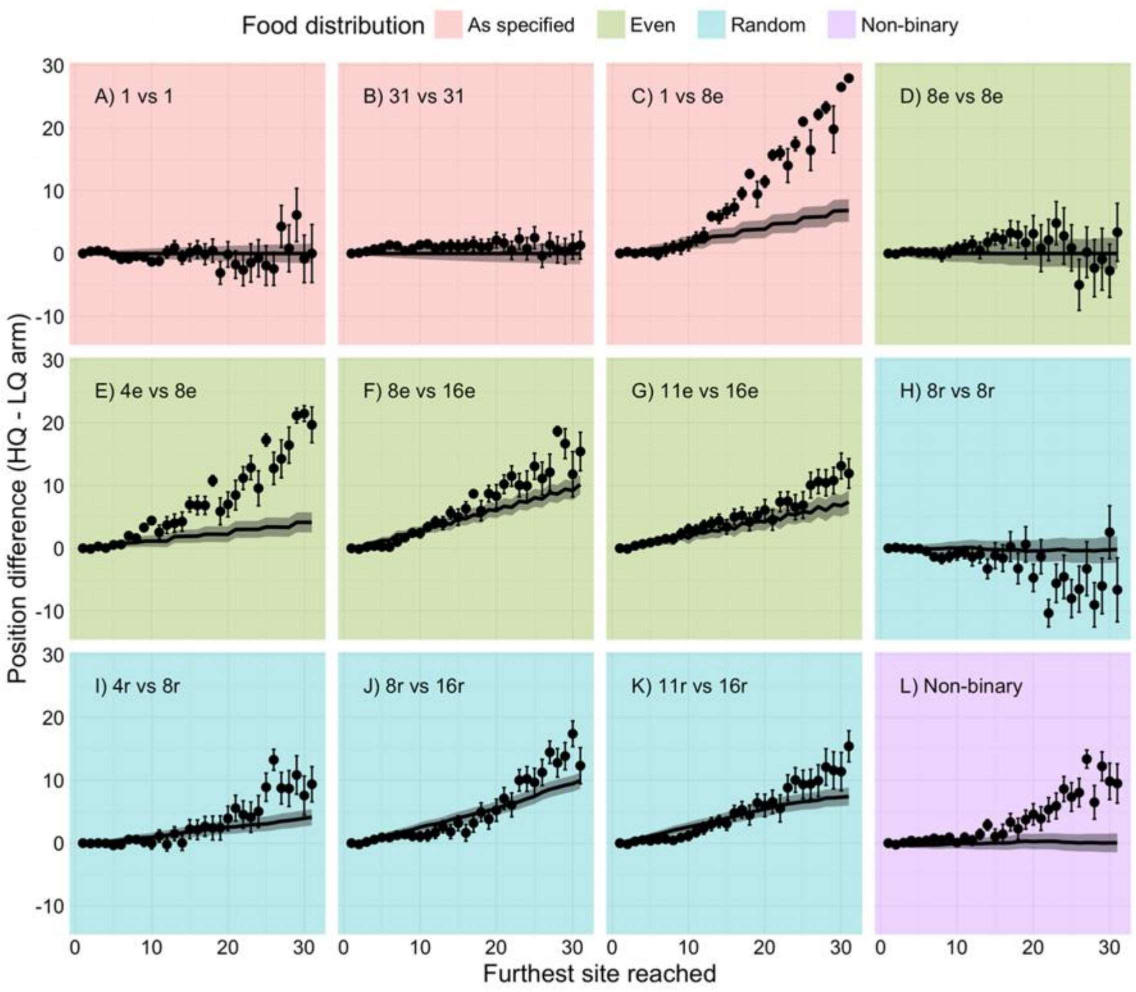

Fig. S12: Relative performance of ‘‘Chemotaxis’ heuristic.

<u>References</u>

1. Jeffreys H. Theory of Probability. Oxford: Oxford University Press; 1939.
